# Condition and Sperm Characteristics of Perch P*erca fluviatilis* inhabiting Boreal Lakes Receiving Metal Mining Effluents

**DOI:** 10.1007/s00244-020-00752-9

**Published:** 2020-07-21

**Authors:** Juha Karjalainen, Hanna E. Arola, Jaana Wallin, Ari Väisänen, Anna K. Karjalainen

**Affiliations:** 1 Department of Biological and Environmental Science, University of Jyväskylä, P.O. Box 35, 40014 Jyväskylä, Finland; 2 Department of Chemistry, University of Jyväskylä, P.O. Box 35, 40014 Jyväskylä, Finland

## Abstract

One of the world’s largest, but low-grade, sulfide nickel deposits in northeastern Finland has been exploited by a bioheapleaching technology since 2008. Bioheapleaching is a relatively new, cost-effective technology, but humid climate, e.g., in boreal temperate environments, causes challenges to the management of the water balance in the ore heaps with wide catchment area, and the mining effluents have caused substantial metal and salting contamination of the receiving waterbodies. In our study, the impacts of metal-extracting bioheapleaching mine effluents on muscle and liver element concentrations, body condition, liver and testes mass, and sperm count and motility of male perch *Perca fluviatilis* were analysed. Liver, testes, and carcass mass of perch in relation to their length were lower in the mining-impacted lakes than in the reference lake, which may be due to the metal contamination, food availability, and energy demand under multistressor conditions. The sperm counts of the males in the mining-impacted lakes were lower, but the endurance of their sperm motility was longer than the endurance of sperm of the reference males. These findings suggested that the condition and sperm characteristics of perch were altered in lakes receiving metal mining effluents. Measured variables seem to be useful indicators for metal mining impacts on freshwater fish but only if high natural variation in these characteristics can be controlled by multiyear monitoring scheme.

One of the world’s largest, but low-grade, sulfide nickel deposit located in Talvivaara, northeastern Finland has been exploited by a bioheapleaching technology since 2008 (Riekkola-Vanhanen 2013). The mining effluents have caused substantial metal and salting contamination of the receiving waterbodies (Salmelin et al. 2017). Bioheapleaching is a relatively new, cost-effective technology, but in boreal temperate environments, the high precipitation causes challenges to the management of the water balance in the ore heaps with very large surface area (Riekkola-Vanhanen 2013). In late 2012, due to the rainy summer and autumn, the gypsum pond leakage at the mine caused severe increase in metal concentrations and salinity of the receiving water bodies with concentrations that are known to be harmful for aquatic biota (Arola et al. 2017). There is evidence from one of the mining-impacted (MI) lakes that the mining effluent from the bioheapleaching process induced water-quality deterioration and has decreased the species richness and diversity of cladoceran and diatom communities (Leppänen et al. 2017).

In different fish species, lower growth rates (Eastwood and Couture 2002; Rajotte and Couture 2002), condition factors (Eastwood and Couture 2002; Levesque et al. 2002; Rajotte and Couture 2002), and hepatosomatic indexes (Rajotte and Couture 2002) have been observed in fish inhabiting metal polluted waters with increased tissue metal concentrations (Rajotte and Couture 2002). Furthermore, metal contamination may lead to higher energetic costs for fish (Sherwood et al. 2000) and, for example, impair the function of the olfactory sensory neurons affecting the antipredatory behavior and feeding of fish (Dew et al. 2014). Metal pollution can deteriorate physiological condition of fish in many ways (Rajotte and Couture 2002) and reduce the genetic diversity within a fish population and the population’s ability to withstand future environmental changes (Bourret et al. 2008). Conversely, Durrant et al. (2011) proposed that convergent adaptive evolutionary processes might lead to metal resistance in a number of geographically distinct brown trout (*Salmo trutta*) populations in southwest England.

Many metals, e.g., copper (Cu), zinc (Zn), cadmium (Cd), and iron (Fe), accumulate mainly in the liver of fish, irrespective of the uptake route (Jezierska and Witeska 2006; El-Moselhy et al. 2014). In most cases, the lowest concentrations of metals in fish are in muscle, whereas metal levels in the liver rapidly increase during exposure and remain high for a long time during depuration when other organs are already cleared (Jezierska and Witeska 2006; El-Moselhy et al. 2014). El-Moselhy et al. (2014) showed that fish exhibited wide, interspecific variations in metals accumulation in all organs. Significant seasonal effects on tissue metal concentrations in fish also have been detected (Couture et al. 2008). Metal concentrations in fish usually follow the ranking: Fe > Zn > lead (Pb) > Cu > Cd > mercury (Hg), but environmental factors affect the uptake and accumulation of metals in fish (Jezierska and Witeska 2006). Metabolic turnover and growth rate affects the accumulation of metals (Honda et al. 1983). Furthermore, water acidification also effects substantially the metal bioaccumulation by changing the solubility of metal compounds (Jezierska and Witeska 2006). Low pH and low alkalinity correlate with high concentrations of metals in liver and dissolved organic material reduces the bioavailability by complexing metals (Eastwood and Couture 2002).

Gonadosomatic indexes of both male and female fish also can be lower in metal-polluted environments (Pyle et al. 2005). Sperm exposure to metals, such as Cd, Cu, Hg, or Pb (Kime et al. 1996; Rurangwa et al. 1998; Lahnsteiner et al. 2004), is known to impair the sperm motility and/or alter the sperm velocity. However, the impact of decreased sperm motility on fertilization success may become evident only when the sperm-to-egg ratio is lowered to a suboptimal level, whereas with sufficient sperm-to-egg ratio, the impact on fertilization success can be masked by the total number of motile sperm (Rurangwa et al. 1998).

Thus, in the spring of 2013 and 2014, we investigated whether mature male perch (*Perca fluviatilis*), living in the lakes (MI lakes) receiving the effluents from the bioheapleaching process from nickel mining, showed signs of physiological stress related to the mining effluent contamination. We evaluated whether their condition and reproductive potential was affected by the mining effluent loading. Condition of fish was analysed by measuring the wet mass of carcass and liver in relation to the total length of fish. Potential effects on reproduction were studied by measuring the wet mass of testes, sperm count, and motility. We also measured element concentrations in the muscles and livers of the male perch.

## Materials and Methods

### Study Lakes

**Fig. 1:**
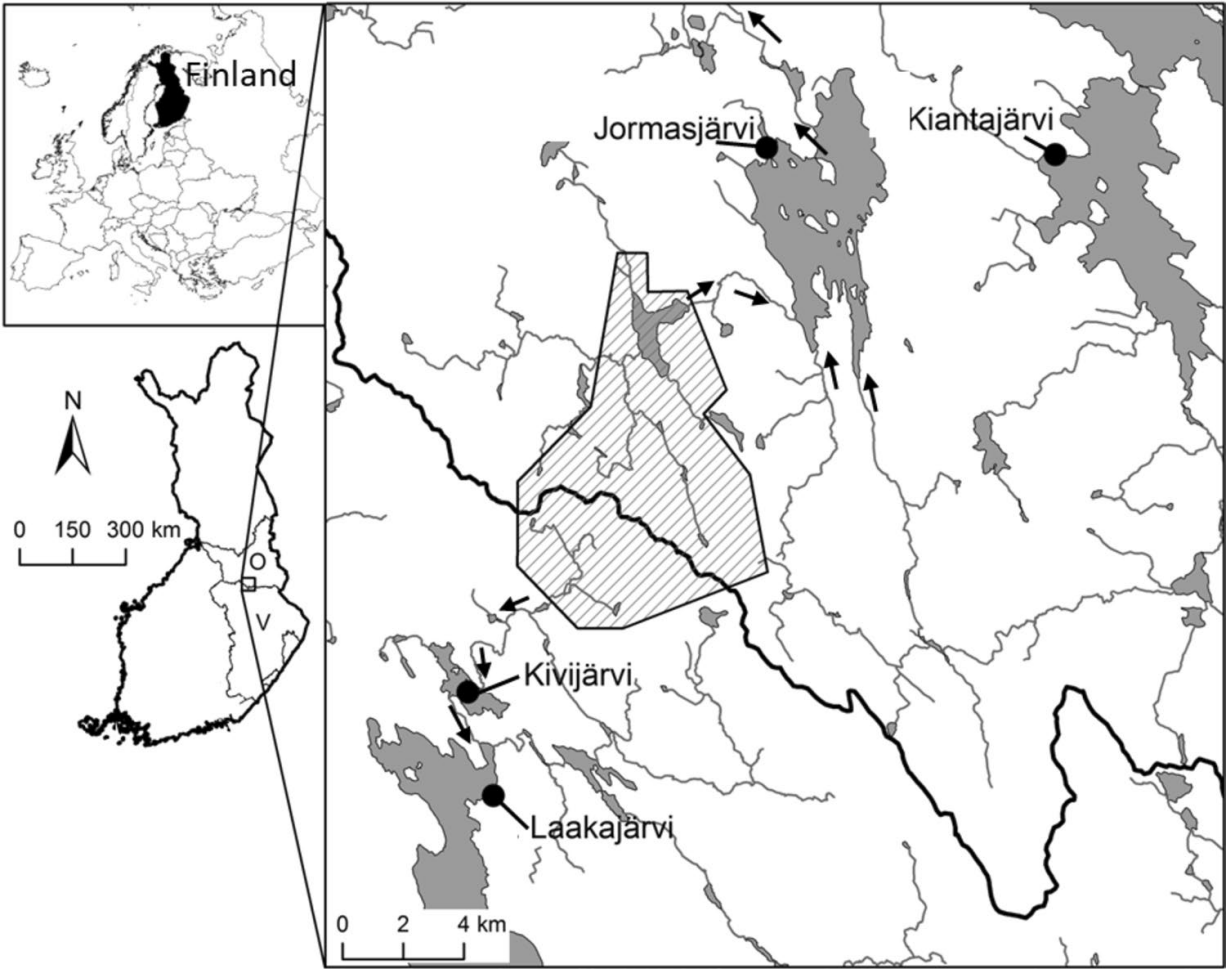
Location of the reference (Kiantajärvi) and mining impacted study lakes (Kivijärvi, Laakajärvi and Jormasjärvi) in Oulujoki (O) and Vuoksi (V) watersheds in Finland. The Talvivaara mine district is indicated in diagonal fill and lakes and streams in grey. Direction of water flow from the mine district to study lakes is shown by arrows. Map data: National Land Survey of Finland, National Database of Regional Land Use Plans, Finnish Environment Institute

Three of the four selected study lakes (lakes Jormasjärvi, Kivijärvi, and Laakajärvi) have received effluent loading from the Talvivaara/Terrafame Sotkamo Mine (Fig. 1). The Talvivaara Sotkamo Mine is located at the border of the watersheds of Oulujoki and Vuoksi, and the mining effluents were discharged into both watersheds. Jormasjärvi is a medium-sized humic lake, whereas Kivijärvi and Laakajärvi are humus-rich lakes, forestry being the main land use type in the catchment areas of all those three lakes (Finnish Environment Institute Database 2017). Of the three lakes, Kivijärvi was the most severely impacted and its ecological status was classified as “bad” (Finnish Environment Institute Database 2017). Both Jormasjärvi and Laakajärvi were moderately impacted by the mining effluents and their ecological statuses were classified as “good” (Finnish Environment Institute Database 2017). The reference lake Kiantajärvi is a medium-sized humic lake with a good ecological status, forestry being the main land use type in its catchment area (Finnish Environment Institute Database 2017).

### Water Quality and Sediment

Epilimnion water temperature, dissolved oxygen, pH, and specific conductance of the sampling sites was measured with YSI6600 multiparameter sonde each time when sample fish were collected. Filtered water samples for dissolved element determination were collected once from each lake in both years. The samples were collected from the shore approximately from 0.2-m depth and filtered (0.45 µm, Whatman PVFD w/PP) into metal-free plastic tubes and acidified immediately with 0.5 mL of nitric acid in the field. The water samples were stored at the dark at + 4 °C until analysis of cadmium (Cd), iron (Fe), manganese (Mn), nickel (Ni), and zinc (Zn) by inductively coupled plasma–optical emission spectrometry (ICP-OES, Optima 8300, Perkin-Elmer). The limits of quantification (LOQ) for each element were defined according to US-EPA (2001). The relative standard deviation limit (RSD) was 10% for all the elemental measurements. Samples were analysed in 2013 and 2014. The water quality data collected in this study was complemented with water quality data for the sampling years 2000‒2014 from the Finnish Environment Institute Water Quality Database (2017). Water samples for dissolved organic carbon (DOC) analysis were collected into glass bottles and stored at the dark at + 4 °C until analysed with Shimadzu Carbon Analyser. Surface sediment samples (0‒5 cm) were taken with a Sandman gravity corer from the sampling site accumulation basins (Wallin 2018). Eight to nine replicate sediment cores were combined to one homogenous bulk sample immediately on the sampling site. The sediment samples were stored at the dark at + 4 °C until analysis. Approximately 0.5 g of dried sediment was weighed into 50-mL plastic tubes. Samples were sonicated (ELMA Transonic 820/H or Bandelin Sonorex RK 512/H) three to four times for 3 min at 50 °C. After sonication, the sediment samples were filtered (Whatman no. 41), and the extract was diluted 122 to 50 mL with ultrapure water. Sediment samples were analysed for element (Cd, Fe, Mn, Ni, Z) concentrations by ICP–OES (PerkinElmer Optima 8300).

### Perch Sampling Protocol in 2013 and 2014

Mature male perch were caught with fish traps (Weke traps, Weke-Katiskat Oy, https://www.weke.fi/en/) in May 2013 and 2014. In 2013, the perch were caught in the beginning of the spawning season in order to obtain representative sperm samples. In 2014, the sperm variables were not analysed, and perch were caught before the spawning season in May. In 2013, total length and wet mass of carcass (body excluding the liver, testes, and alimentary canal), liver, and testes of the perch were measured. Also, the sperm count, motility, and velocity, muscle, and liver element concentrations were analysed in 2013. Perch were transported to the field laboratory in aerated plastic water tanks containing their natal lake water, killed with a blow to the head, and measured at the field laboratory.

In 2014, only total length and wet mass of carcass, liver, and testes of the perch were measured. The perch were killed with a blow to the head and measured either in the field laboratory immediately after catching, or stored at − 20 °C and measured later. The frozen perch were thawed at + 4 °C or in a water bath in plastic bags and measured thereafter. Number of fish in sampling years and lakes and their sizes and ages are given in Table 1. Age of the fish was determined from the scales.

**Table 1:** Total length (cm, mean ± standard error SE), total wet mass (g, mean ± SE), wet mass of testes (g, mean ± SE), wet mass of carcass (g, mean ± SE), wet mass of liver (g, mean ± SE), and median (minimum–maximum) age (years) of sampled fish in 2013 and 2014 in the reference lake (Kianta) and the mining impacted lakes

	Kianta 2013	Jormas 2013	Laaka 2013	Kivi 2013	Kianta 2014	Jormas 2014	Laaka 2014	Kivi 2014
Total length	17.0 ± 0.5 (14)	16.5 ± 0.4 (26)	13.1 ± 0.5 (10)	13.8 ± 0.5 (16)	17.5 ± 0.4 (36)	15.3 ± 0.5 (53)	12.6 ± 0.2 (20)	13.7 ± 0.2 (79)
Total mass	47.4 ± 4.5 (14)	41.2 ± 2.8 (26)	18.2 ± 2.4 (10)	25.2 ± 2.9 (16)	59.5 ± 3.6 (36)	39.2 ± 3.3 (53)	18.0 ± 0.7 (20)	23.7 ± 1.1 (79)
Testes mass	2.4 ± 0.2 (14)	1.8 ± 0.2 (17)	0.5 ± 0.1 (10)	0.7 ± 0.1 (16)	2.4 ± 0.2 (36)	1.4 ± 0.2 (53)	0.8 ± 0.1 (19)	0.8 ± 0.1 (77)
Carcass mass	43.1 ± 4.1 (14)	37.4 ± 2.9 (17)	23.6 ± 2.8 (10)	17.0 ± 2.2 (10)	53.7 ± 3.3 (36)	35.6 ± 3.0 (53)	21.3 ± 1.0 (79)	16.2 ± 0.7 (20)
Liver mass	0.5 ± 0.2 (5)	0.4 ± 0.03 (17)	0.13 ± 0.02 (10)	0.19 ± 0.03 (16)	0.78 ± 0.1 (36)	0.50 ± 0.1 (53)	0.17 ± 0.02 (20)	0.19 ± 0.01 (79)
Age	3 (3–5)	5 (3–6)	4 (3–6)	4 (3–6)	4 (2–5)	4 (2–6)	4 (3–6)	4 (3–6)
Fish in sperm analysis	14	24	10	13	–	–	–	–

Number of fish measured is represented in parentheses. Number of males in the sperm analysis is given in the last row

–No samples taken for sperm analysis in 2014

### Tissue Element Concentrations

Fish were kept on ice during the dorsal muscle and liver sampling, and the samples were stored in plastic bags at − 20 °C until analysed. For the tissue metal concentration analyses, the frozen samples were thawed at room temperature, weighed, and dried at + 105 °C for 24 h. For the analyses, dried muscle and liver (61–94 mg and 8–78 mg, respectively) were weighed into plastic tubes. The tissue samples were digested by moistening the samples with a few drops of ultrapure water, adding 3 mL (sample dry weight > 40 mg) or 1.5 mL (sample < 40 mg) of aqua regia (HNO_3_:HCl, 1:3, v:v) into each sample and sonicating the samples for 3 min at 45–60 °C (De La Calle et al. 2009). The digested samples were filtered (Whatman 41) and filled up to the final volume of 20 mL (sample dry weight > 40 mg) or 10 mL (sample dry weight < 40 mg) with ultrapure water. The concentrations of cadmium (Cd), iron (Fe), manganese (Mn), nickel (Ni), sulfur (S), and zinc (Zn) were analysed with ICP-OES (Optima 8300, Perkin-Elmer), and the LOQs and RSD requirements were the same as for the water samples. Additionally, the muscle Ni concentrations were analysed with electrothermal atomic absorption spectrometer (ETAAS, Model Aanalyst 800 equipped with an AS-800 autosampler, Perkin-Elmer) in order to reach lower analysis LOQs of 0.31 µg L^−1^ for Ni. Concentrations ≤ LOQ (mg L^−1^) or with RSD > 10% were discarded from the calculations. In muscle tissue, 2% of Fe, 2% of S, 23% of Ni, and 73% of Mn samples were discarded because of LOQ and RSD limits. Cd concentrations in muscle and Ni concentration in liver was largely under LOQ, and thus, all samples were excluded. The quality assurance of the method was performed by the analysis of blank samples and certified reference materials TORT-2 (Lobster hepatopancreas) and DOLT-4 (Dogfish liver) supplied by the National Research Council (NRC). Samples were analysed in 2013 and 2014. We reported in this study the Cd, Fe, Mn, Ni, Zn, and S results, because these elements are the main components in the ore effluents.

### Sperm Analyses

Sperm counts, velocity, and proportion of motile sperm of males from reference lakes and from the impacted lakes were analysed with Proiser ISASv1^®^ CASA system. The sperm counts represent one random microscopic field of vision. The motility was measured as the velocity of a sperm head along the total trajectory (VCL, µm s^−1^), and the velocity as the velocity of a sperm head along a straight line between its first detected position and its last detected position (VSL, µm s^−1^) (Quintero-Moreno et al. 2003).

The milt was collected into microcentrifuge tubes or taken directly into a plastic pipette tip (10 µL) immediately after killing the individual. The milt temperature was kept at 9–10 °C by the Echtotherm Chilling dry bath. CASA analysis was done without delay fish by fish sequentially. A drop of undiluted milt was smeared on the opening of the sperm counting slide (LEJA, ~ 6 µL), 5 µL of the activation water was added, and sperm variables were recorded 10 and 20 s after the activation. Three different activation waters were used: artificial freshwater (0.1 mmol L^−1^; Anonymous, 1996); cadmium solution (50 mg Cd L^−1^, CdCl_2_, anhydrous, dissolved into the artificial freshwater); and filtered (48-µm mesh) lake surface water from the natal lake of the fish. Sperm variables with each activation water types were measured in three replicates for each male, and a mean of the three measurements was used for the further analysis. The activation water temperature was 9–10 °C, but during the sperm measurements, the temperature of the slide was not controlled.

### Data Analyses

A lake-wise ANOVA was used in analyzing the mining impact on liver and muscle tissue metal concentrations. Liver concentrations of Fe, Zn, Mn, Cd, and S and muscle concentrations of Fe, Zn, Mn, Ni, and S were selected for the dependent variables, since the concentrations of those metals are major components of the ore effluents and also SO_4_ have been elevated in the MI lakes (Kauppi et al. 2013). All of the variables were log_10_-transformed for the analyses. All multiple comparisons were based on the estimated marginal means with the Least significant difference (LSD) procedure. The impact of the fish body size on the tissue element concentrations was tested as well by inserting the log_10_-transformed wet mass of the carcass as a covariate, but because its effect was insignificant on all the variables, it was excluded from the final analyses. Homogeneity of variance was observed in all models of the elements (Levene, *p *> 0.05). All log_10_-transformed variables of the elements were normally distributed (Shapiro–Wilk, *p* > 0.05). Pearson correlation was used to analyze correlation between log_10_-trasformed element concentrations, and liver and testes masses from the pooled data of all lakes together.

An ANCOVA was used in analyzing the mining impact on the condition of male perch between lakes. The dependent variables were wet mass (g) of carcass (excluding alimentary canal, liver, and testes), liver, and testes, which all were log_10_-transformed for the analyses. To analyze the carcass-mass-at-length of the fish, the log_10_-transformed total length of perch was set as a covariate and the sampling year and lake as the independent factors. Transformation was due to nonlinear relationships between the total length and the masses. All pairwise comparisons were made based on the estimated marginal means with the LSD procedure. Only individuals with wet body mass below 100 g were included into the measurements and analyses in order to harmonize the fish size between lakes. Homogeneity of variance was tested by the Levene test, and the normality test was done by Shapiro–Wilk test. Homogeneity of variance was observed in all models (Levene, *p *> 0.05), except in the analysis of carcass mass with log_10_-transformation (Levene, *p* = 0.01). We interpreted that this minor discrepancy in assumption underlying in the models did not disturb our overall interpretation. All three log_10_-transformed variables (carcass mass, testes, liver) were normally distributed (Shapiro–Wilk, *p* > 0.05).

The mean individual total sperm count was calculated from the mean sperm count of all activation water types at both 10 and 20 s measuring frame. An ANOVA was used in analyzing the mining impact on the sperm count (no. of sperm per a microscope frame). All pairwise comparisons were made based on the estimated marginal means with a LSD procedure. Also, the relation between the size of the male (total length) as well as the proportion of motile sperm on the total sperm count was inspected. Log_10_-transformed total length was used firstly as a covariate in the ANCOVA but was excluded from the final ANOVAs, because the effect of total length was not statistically significant in the models. The correlations between the sperm count and the wet mass of the males or the sperm count and the proportion of active sperm in samples were analyzed by Spearman’s correlation coefficient.

To inspect the differences in sperm motility among the different activation waters, a repeated measures ANOVA and Bonferroni multiple comparison procedure was used: within-subject factor was the activation water (Cd solution, artificial freshwater, and lake water) and between-subject factor sampling time (10- and 20-s sampling). The sperm motility (proportion of motile sperm with arcsine-transformation) and velocity (VSL) differences among the males from the different lakes were analysed only from the lake water activated sperm and separately for both 10- and 20-s measurements. Static spermatozoa were excluded from the velocity analyses. A lake-wise ANOVA was used in analyzing the mining impact on the proportion of motile sperm and sperm velocity. All pairwise comparisons were made based on the estimated marginal means with LSD procedure. Homogeneity of variance was observed in both models (Levene, *p *> 0.05), except in the analysis of proportion of motile sperm with arcsine-transformation at 10 s (Levene, *p* = 0.027). This minor discrepancy in assumption underlying in the models was accepted. Both sperm motility and velocity was normally distributed (Shapiro–Wilk, *p* > 0.05). All of the statistical analyses were made with IBM SPSS 24.

## Results

### Water Quality

**Fig. 2:**
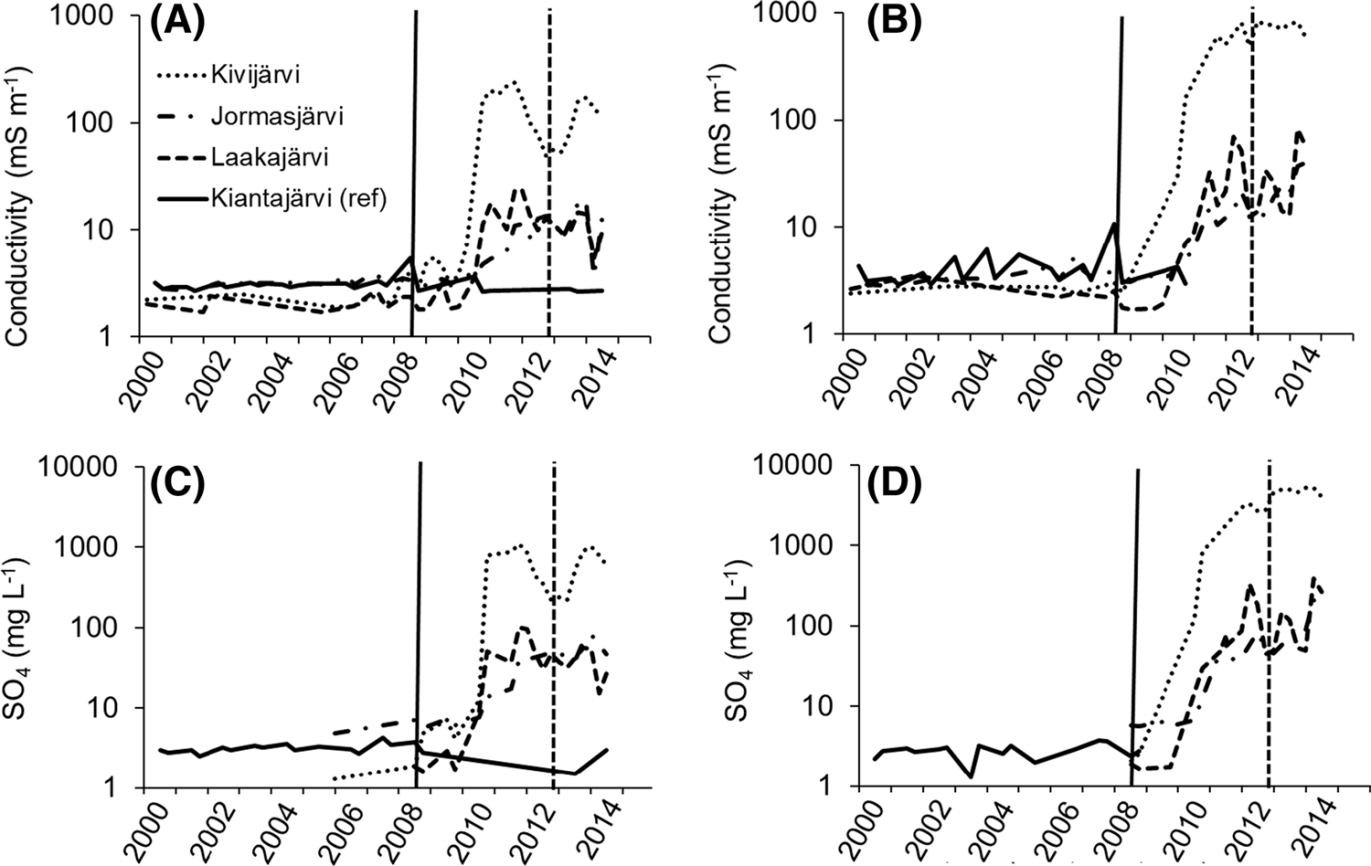
Mean seasonal conductivity and SO_4_ concentration from spring 2000 to spring 2014 in the epilimnion (**a** and **c** depth layer 0–2 m from the surface) and hypolimnion (**b** and **d**, depth layer 0–2 m above the lake bottom) in Kiantajärvi (reference lake), Jormasjärvi, Laakajärvi and Kivijärvi. The solid vertical line indicates the beginning of the mining operations in spring 2008 and the dashed line the gypsum pond leakage at the mine in autumn 2012. Data sources from Finnish Environment Institute Water Quality Database (2017). Hypolimnion data for Kiantajärvi are not available for whole monitoring period

Surface water temperature, dissolved oxygen, pH, and specific conductance of the sampling sites during the catching fish was given in Table 2. The long-term trends in the water quality (database of Finnish Environment Institute and own measurements) showed that the electric conductivity of the lake water started to increase in the MI study lakes, Jormasjärvi, Laakajärvi, and Kivijärvi, roughly from 2010 onwards after the beginning of the mining in 2008 (Fig. 2a epilimnion, b hypolimnion). Of these three lakes, especially in Kivijärvi, the conductivity increased rapidly after 2010. Simultaneously, in the three MI lakes, the seasonal SO_4_ concentrations in both the epilimnion and hypolimnion increased (Fig. 2c, d). The highest seasonal mean concentrations of dissolved Fe, Zn, Mn, Cd, and Ni were observed in the hypolimnion of Kivijärvi, and these elements were elevated in the sediment of the MI lakes compared with the reference lake (Table 3).

**Table 2:** Mean water quality of surface water (< 1 m depth) (mean, *n* = 1–3) of the study lakes in spring 2013 and 2014: water temperature (°C), pH, oxygen concentration (mg L^−1^), oxygen saturation (%), dissolved organic compounds (DOC, mg L^−1^)

	Kianta 2013	Jormas 2013	Laaka 2013	Kivi 2013	Kianta 2014	Jormas 2014	Laaka 2014	Kivi 2014
Temperature	14.3	14.1	11.0	16.6	8.0	6.9	6.8	7.3
pH	6.4	6.4	5.8	6.4	6.6	6.1	5.5	6.4
Oxygen	11.0	11.0	9.8	9.2	11.5	11.2	10.6	10.5
Oxygen %	106.5	106.3	88.8	93.8	96.2	92.0	87.0	87.1
DOC	10.5	9.2	9.2	8.0	10.7	9.2	13.7	10.5

**Table 3:** Mean concentrations (± standard error) of Fe, Zn, Mn, Cd, and Ni in the epilimnion (< 1 m depth) and hypolimnion (1 m from the bottom) in the study lakes in 2010–2014 and in the sediment in 2015

Zone	Lake	Fe mg L^−1^	Zn µg L^−1^	Mn mg L^−1^	Cd µg L^−1^	Ni µg L^−1^
Epilimnion	Kianta	0.59 ± 0.03	< LOQ	0.11 ± 0.02	–	12.65 ± 0.05
	Jormas	0.55 ± 0.08	33.45 ± 5.29	0.07 ± 0.01	0.09 ± 0.01	12.34 ± 1.69
	Laaka	0.80 ± 0.25	11.77 ± 2.36	0.37 ± 0.12	0.03 ± 0.01	5.91 ± 1.67
	Kivi	0.62 ± 0.10	29.38 ± 4.16	4.53 ± 1.21	0.09 ± 0.03	32.49 ± 4.54
Hypolimnion	Kianta	0.59 ± 0.04	< LOQ	–	–	12.7*
	Jormas	0.88 ± 0.17	57.70 ± 11.91	0.70 ± 0.21	0.14 ± 0.01	15.5 ± 1.09
	Laaka	0.75 ± 0.06	11.99 ± 2.23	1.19 ± 0.52	0.07 ± 0.03	11.4 ± 2.18
	Kivi	61.93 ± 15.87	155.27 ± 83.18	58.83 ± 16.57	0.44 ± 0.22	373.20 ± 153.95

Zone	Lake	Fe mg g^−1^	Zn mg g^−1^	Mn mg g^−1^	Cd mg kg^−1^	Ni mg g^−1^
Sediment	Kianta	18.14 ± 2.04	0.09 ± 0.01	1.26 ± 0.13	1.43 ± 0.37	0.02 ± 0.01
	Jormas	38.09 ± 1.78	0.46 ± 0.04	1.94 ± 0.19	5.52 ± 0.51	0.10 ± 0.01
	Laaka	32.44 ± 2.86	0.14 ± 0.02	2.01 ± 0.39	3.25 ± 0.62	0.03 ± 0.01
	Kivi	23.26 ± 1.67	0.31 ± 0.05	2.15 ± 0.44	2.37 ± 0.52	0.21 ± 0.02

Under the limits of quantification results are indicated by < LOQ. Data sources for the elements: Finnish Environment Institute Water Quality Database (2017) and own measurements in 2013, 2014, and 2015

–Not analysed

*n = 1, no standard error

### Tissue Element Concentrations

The Fe, Mn, and Cd concentrations in the perch liver were significantly different among lakes (Table 4). The Fe concentration of the perch livers in the reference lake Kiantajärvi differed significantly from the Fe concentrations in the MI lakes and the Fe concentrations increased with severity of MI on the lakes. In Kivijärvi, the Mn concentration of perch livers differed from the Mn concentrations in Kiantajärvi and Jormasjärvi. The lake-wise comparisons revealed that the males from Jormasjärvi and Kivijärvi had significantly higher liver Cd concentrations than the perch in Kiantajärvi and Laakajärvi. The highest Cd concentrations were not found in the most significantly impacted lake. The liver Zn and S concentrations did not differ significantly between the lakes (Table 4).

**Table 4:** Mean element concentrations (± standard error, number of cases in parentheses) mg kg^−1^ (dry weight) in the livers of the male perch caught 2013

Lake	Fe	Zn	Mn	Cd	S
Kiantajärvi	^a^264 ± 24 (*n* = 4)	130 ± 10.9 (*n* = 4)	^a^9.37 ± 0.95 (*n* = 4)	^a^2.13 ± 0.45 (*n* = 4)	8258 ± 726 (*n* = 4)
Jormasjärvi	^b^585 ± 63 (*n* = 15)	147 ± 8.3 (*n* = 15)	^a^9.55 ± 0.58 (*n* = 15)	^c^23.4 ± 2.07 (*n* = 15)	8732 ± 330 (*n* = 15)
Laakajärvi	^bc^905 ± 183 (*n* = 2)	127 ± 5.8 (*n* = 2)	^b^14.65 ± 4.31 (*n* = 2)	^a^3.87 ± 1.03 (*n* = 2)	9857 ± 739 (*n* = 2)
Kivijärvi	^c^1412 ± 267 (*n* = 5)	157 ± 10.9 (*n* = 5)	^b^15.01 ± 1.19 (*n* = 5)	^b^12.34 ± 2.74 (*n* = 5)	10,195 ± 620 (*n* = 5)
ANOVA *F* *p*	16.800 < 0.001	0.906 0.454	6.122 0.003	36.112 < 0.001	2.183 0.119

Observations below the LOQ were discarded from the calculations. ANOVA *F* and statistical significances (*p*) are given in the last row of the table. Superscript letters before the means represent statistically significant differences between the lakes (LSD pairwise comparison, different letters indicate statistically significant difference, *p* < 0.05). Statistical analyses were made with log_10_-transformed values

There were no significant differences in muscle Fe, Zn, and Ni concentrations among the lakes (Table 5). The significantly different muscle S concentrations among the lakes (Table 5) showed that perch in Kivijärvi had significantly higher concentrations of S in their muscle than perch in the reference lake Kiantajärvi and Jormasjärvi.

**Table 5:** Mean element concentrations (± SE, number of cases in parentheses,) mg kg^−1^ (dry weight) in the muscles of the male perch caught 2013. Observations below the LOQ were discarded from the calculations

	Fe	Zn	Mn	Ni	S
Kiantajärvi	14.59 ± 4.84 (*n* = 5)	26.80 ± 3.88 (*n* = 5)	–	1.13 ± 0.16 (*n* = 4)	^a^9782 ± 744 (*n* = 5)
Jormasjärvi	19.27 ± 4.22 (*n* = 16)	26.08 ± 4.41 (*n* = 17)	1.19 ± 0.56 (*n* = 5)	1.14 ± 0.19 (*n* = 16)	^a^9983 ± 352 (*n* = 17))
Laakajärvi	17.46 ± 3.9 (*n* = 10)	20.82 ± 2.35 (*n* = 10)	9.79 ± 4.82 (*n* = 5)	0.98 ± 0.54 (*n* = 5)	^ab^10944 ± 531 (*n* = 9)
Kivijärvi	17.38 ± 2.01 (*n* = 16)	21.17 ± 2.22 (*n* = 16)	1.55 (*n* = 1)	1.38 ± 0.23 (*n* = 11)	^b^11685 ± 258 (*n* = 16)
ANOVA *F* *p*	0.487 0.693	0.543 0.655	–	0.800 0.503	4.935 0.005

ANOVA *F* and statistical significances (*p*) are given in the last row of the table. Superscript letters before the means represent statistically significant differences between the lakes (LSD pairwise comparison, different letters indicate statistically significant difference, *p* < 0.05). Statistical analyses were made with log_10_-transformed values

–Not analyzed

The Fe concentration of the liver correlated negatively with the wet mass of liver and testes (Pearson *r* = − 0.551, *p* = 0.004, *n* = 26 and *r* = − 0.629, *p* = 0.001, *n* = 26). Also, the muscle Fe concentration correlated negatively with the liver mass (*r* = − 0.303, *p* = 0.038, *n* = 47) but not with the testes mass (*r* = − 0.170, *p* = 0.255, *n* = 47). The muscle S concentration correlated negatively with the wet mass of liver and testes (Pearson *r* = − 0.307, *p* = 0.036, *n* = 47 and *r* = − 0.356, *p* = 0.014, *n* = 47). The Cd, Mn, and Zn concentrations of both tissues and the liver S concentration did not correlate with the liver or testes mass (*p* > 0.05).

### Condition of Perch

**Fig. 3:**
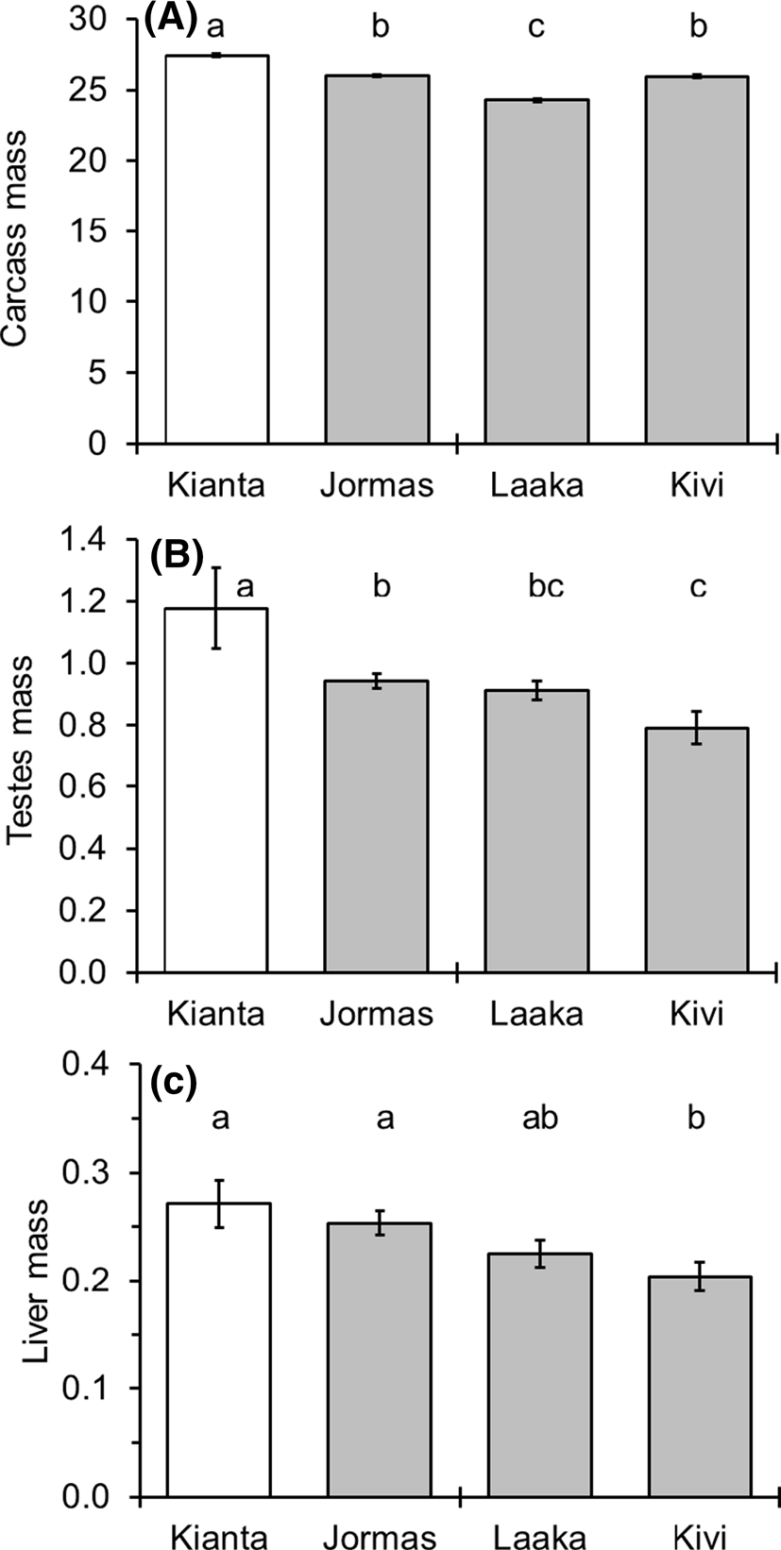
Estimated marginal means wet mass (g) of carcass (**a**), testes (**b**), and liver (**c**) of perch male caught from the reference (open bar) and mining impacted lakes in 2013 and 2014. Vertical lines represent 95% confidence intervals and letters above the bars statistically significant differences between the lakes (LSD paired comparison, different letters indicate statistically significant difference, *p* < 0.05)

The wet masses of carcass differed significantly between the perch males in the study lakes (Fig. 3a, ANCOVA, *F* = 9.363, *df* = (3, 245), *p* < 0.001), the males in the reference lake, Kiantajärvi, which had significantly higher wet mass than males in the MI lakes (LSD pairwise comparison, *p* < 0.05). Both the sampling year and the covariate total length of fish was statistically significant (*p* < 0.001). The interaction between lake and year was significant (*p* < 0.001), because in Kiantajärvi in 2014, the wet mass was higher than in 2013, whereas in the MI lakes the wet mass was similar in both years. The lake-wise difference in testes mass was significant (ANCOVA, *F* = 6.134, *df* = (3, 242), *p* < 0.001), and the total length was a significant covariate (*p* > 0.001) while the year was not (*p *= 0.102). Males from Kiantajärvi had the highest mean wet mass of testes, which was significantly higher than the testes of males from the MI lakes (Fig. 3b, LSD pairwise comparison, *p* < 0.05). The wet mass of liver of the perch males differed significantly among lakes (Fig. 3c, ANCOVA, *F* = 2.719, *df* = (3, 236), *p* = 0.045) and the covariate—total length of fish—also was highly significant (*p* < 0.001). The liver masses of the Kivijärvi perch were lower than the liver masses in the reference lake (LSD pairwise comparison, *p* = 0.024).

### Sperm Counts and Motility

**Fig. 4:**
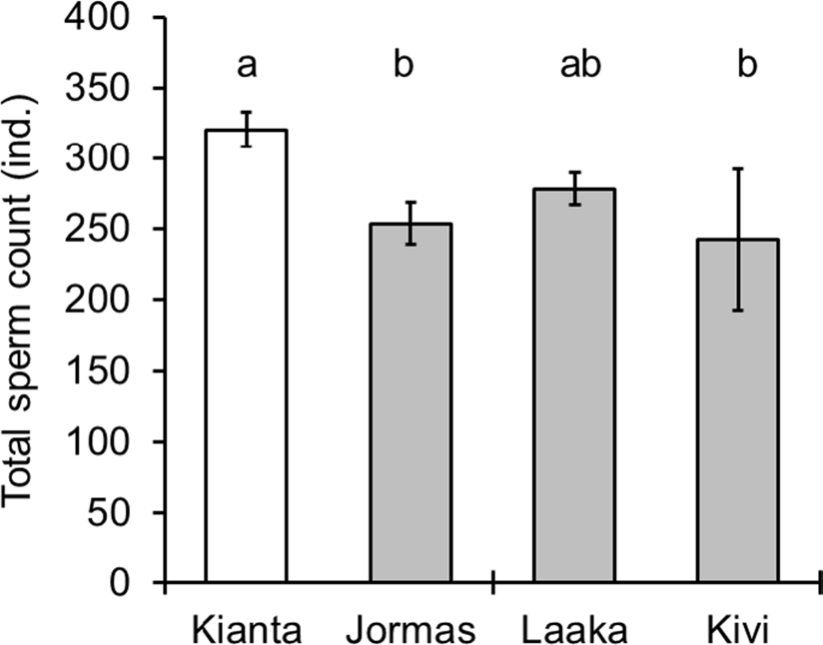
Total mean sperm count (individuals) of perch caught in 2013 from the reference (open) and mining impacted lakes. Vertical lines represent the standard error of mean and letters above the bars statistically significant differences between the lakes (LSD paired comparison, different letters indicate statistically significant difference, *p* < 0.05)

The sperm counts of the perch varied significantly among the lakes (Fig. 4, ANOVA, *F* = 5.244, *df* = (3, 61), *p* = 0.003); the reference lake males had significantly higher sperm counts than males in Jormasjärvi and Kivijärvi (LSD paired comparison, *p* < 0.001). A statistical significance (*p* value) for the difference in sperm count between Kiantajärvi and Laakajärvi by LSD comparison was 0.081. The sperm counts of males between the MI lakes did not differ significantly (LSD pairwise comparison, *p* > 0.1). The sperm count was not in relation to the wet mass of the males (Spearman’s *r *= 0.204, *n* = 61, *p* = 0.116) or the proportion of active sperm in samples (Spearman’s *r* = 0.043, *n* = 61, *p* = 0.740).

The proportion of motile sperm differed significantly among the different types of activation waters (repeated measures ANOVA, *F* = 13.543, *df* = (2, 56), *p* < 0.001). Artificial freshwater and lake water treatments did not differ significantly (Bonferroni, MD = 0.022, *p* = 1.000), but they both had significantly higher proportions of motile sperm compared with the Cd treatment (Bonferroni, MD = 0.205–0.226, *p* ≤ 0.001).

**Fig. 5:**
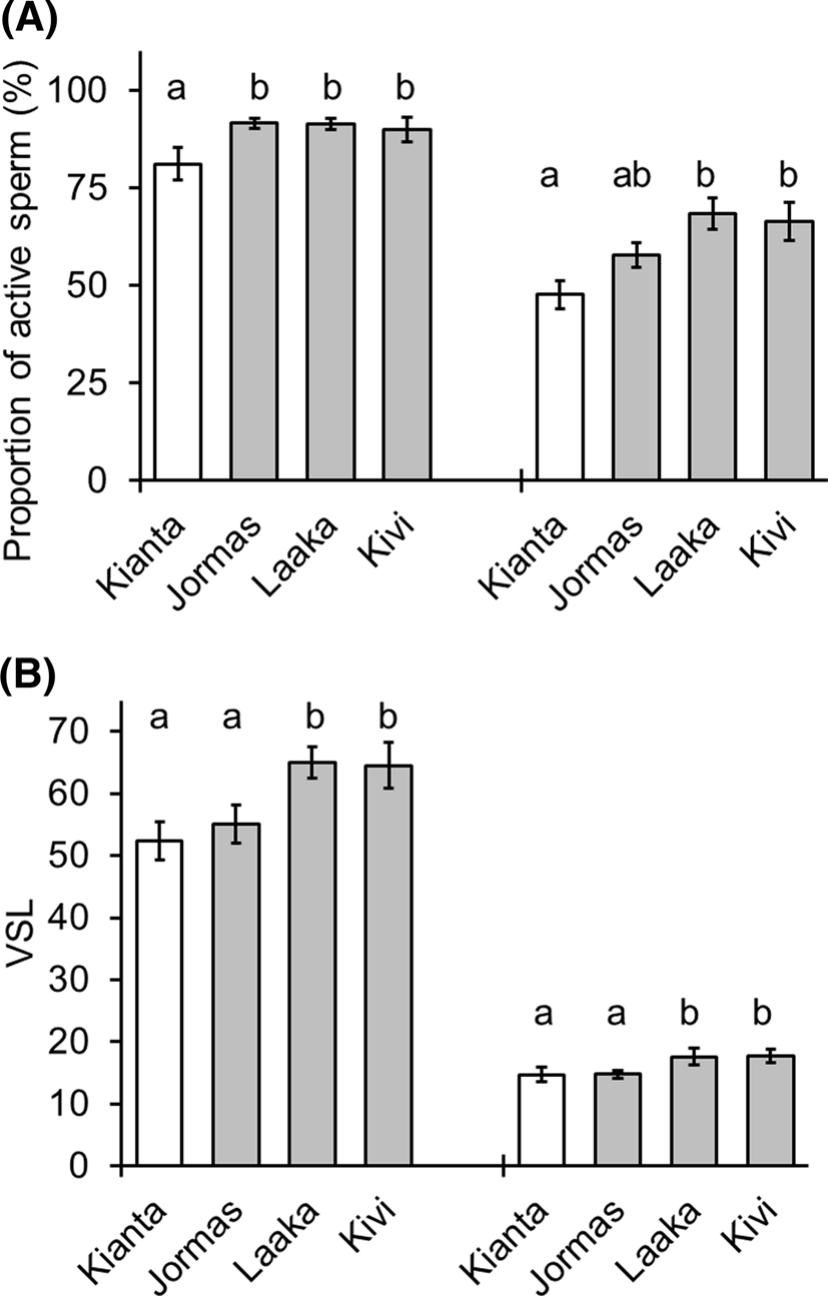
Relative proportion of active sperm (**a**, %) and velocity (**b**, VSL, µm s^−1^) of a sperm head along the straight line between its first detected position and its last position in the sperm activation test of perch with activation by lake water from each fish’s natal lake in the sampling occasions of 10 s (4 bars in left) and 20 s (4 bars in right). Vertical lines represent the standard error of mean and letters above the bars statistically significant differences between the lakes (LSD paired comparison, different letters indicate statistically significant difference, *p* < 0.05)

In the sperm measurements taken 10 s after the sperm activation with the natal lake water of the fish, the lake-wise comparisons showed significant differences in the perch sperm motility among the different lakes (Fig. 5a, ANOVA, *F* = 3.400, *df* = (3, 61), *p *= 0.024). In the reference lake, Kiantajärvi, the perch males had statistically significantly lower proportion of motile sperm compared with males in MI lakes (LSD pairwise comparison, *p* < 0.05). Similarly, the VSL of sperm differed significantly among the lakes (Fig. 5b, ANOVA, *F* = 2.398, *df* = (4, 66), *p* = 0.059) being lower in Kiantajärvi and Jormasjärvi than in Laakajärvi and Kivijärvi (LSD pairwise comparison, *p* < 0.05). In the subsequent sperm measurements taken 10 s later, the proportion of motile perch sperm differed significantly between the lakes (Fig. 5a, ANOVA, *F* = 5.051, *df *= (3, 61), *p *= 0.004). In the Kiantajärvi, the perch males had statistically significantly lower proportion of motile sperm compared with males in Laakajärvi and Kivijärvi (LSD paired comparison, *p* = 0.002). A statistical significance (*p* value) for the difference in sperm motility between Kiantajärvi and Jormasjärvi by a LSD pairwise comparison was 0.059. The VSL of sperm differ significantly among the lakes (Fig. 5b, ANOVA, F = 2.885, *df* = (3, 61), *p *= 0.043): lower in Kiantajärvi and Jormasjärvi than in Laakajärvi and Kivijärvi (LSD pairwise comparison, *p* < 0.05).

## Discussion

The mining effluent induced salinity and metal contamination was evident in the three MI study lakes, particularly in Kivijärvi. Contamination affected the liver tissue concentrations of perch males but in general not the muscle concentrations, except that the S concentration of Kivijärvi was higher than muscle S in Kiantajärvi and Jormasjärvi. In our study lakes, Fe and Zn had the highest concentrations of the measured metals in liver and muscle of perch. Liver Fe concentration in the MI lakes varied from 323 to 2326 mg kg^−1^ and from 5 to 77 mg kg^−1^ in muscle tissue. In other metal polluted lakes, the range of Fe concentrations in liver and muscle of different fish species has been reported to be 50–4020 mg kg^−1^ and 4–100 mg kg^−1^, respectively (Honda et al. 1983; Szarek-Gwiazda et al. 2006; Rajkowska and Protasowicki 2013; El-Moselhy et al. 2014; Mohamed et al. 2016), whereas the Zn concentrations in liver and muscle have been 60–320 mg kg^−1^ and 1–50 mg kg^−1^, respectively (Honda et al. 1983; Tkatcheva et al. 2000; Eastwood and Couture 2002; Rajkowska and Protasowicki 2013; El-Moselhy et al. 2014; Yancheva et al. 2014). In our MI lakes, the Zn range was 108–213 in fish livers and 10–85 in muscles.

Metal exposure has been observed to cause changes in hepatic tissue (Tkatcheva et al. 2000; El-Moselhy et al. 2014; Yancheva et al. 2014; Abalaka 2015; Mohamed et al. 2016). Especially Cd (Tkatcheva et al. 2000), Cu, and Fe exposure (Mohamed et al. 2016) have caused liver cell damage, such as apoptosis, necrosis, fibrosis, and hepatocyte lysis. Jezierska and Witeska (2006) noted in their review that various studies have shown synergistic or additive effects of metals in fish. In our study, no histological analyses of hepatic tissue were performed, but the perch males from the MI lakes had smaller livers than fish caught from the reference lake, suggesting harmful effects of the metal and salinity contamination of these lakes to perch males.

Smaller livers, testes, and lower wet carcass mass in relation to the length of fish may suggest low food availability and/or harmful shifts in prey availability and subsequent higher-energy demand in our MI study lakes under unstable multi-stressor conditions. In an earlier study with golden perch (*Macquaria ambigua*), starvation has been demonstrated to decrease the hepatosomatic index and increase the utilization of liver energy reserves (Collins and Anderson 1995). The diversity of the benthic macroinvertebrate communities can be reduced in metal-contaminated lakes, and the lack of suitable prey for diet shift can increase the activity costs and cause slow growth and even stunting of the fish, as observed with yellow perch (Sherwood et al. 2002; Iles and Rasmussen 2005). Additionally, Sherwood et al. (2000) have demonstrated that the food conversion efficiency to growth has been reduced in yellow perch in metal contaminated lakes. Similarly, Overton et al. (2008) suggested that the observed growth impairment in Eurasian perch exposed to increased water salinity (10%) may have been linked to the reduced allocation of food to growth. Behavior of fish also has showed to be affected by the impairment of their sensory neurons with behavioral deficits under metal contamination (Dew et al. 2014), which may influence seriously their capability to observe their environment and diminish the antipredator behavior, survival, and likely on their growth. In all, in several studies, the increased tissue metal concentrations have been associated with the decrease in the condition and growth of fish (Levesque et al. 2002; Rajotte and Couture 2002).

In our MI study lakes, the significantly lower sperm counts and testes size of the perch males suggested that the mining effluent contamination have disturbed their investments to the reproduction, probably due to repeated chronic multi-stressor conditions in water quality. For example, Campbell et al. (1992) have demonstrated lower sperm densities in rainbow trout (*Oncorhynchus mykiss*) when they have been repeatedly exposed to stressful anoxic treatment. In our study lakes, the lower sperm counts seemed to be compensated by investing in sperm quality, as the proportion of motile sperm and the linear swimming velocity (VSL) of the sperm was higher in the males of the MI lakes than in the males of the reference group. A similar relationship between the sperm density and active sperm has been found with haddock (*Melanogrammus aeglefinus*): when the spermatocrit exceeded 70%, the proportion of motile sperm was decreased (Rideout et al. 2004). The quality of gametes is an important trait affecting the reproductive success and maximizing fitness of fish, and several environmental factors regulate the energy allocation to the reproductive products (Wootton 1998). Hutchings (1991) showed that female brook trout (*Salvelinus fontinalis*) produce small number of large eggs under low food conditions and many small eggs under high food conditions. Our results propose similar association between sperm quantity and quality. In other studies, the gonadosomatic index of male yellow perch (*Perca flavescens*) have been observed to be lowered in metal contaminated lakes (Levesque et al. 2002; Pyle et al. 2005). In these Canadian lakes, the free metal ion or total Cd, Ni, and Zn concentrations were at the same level or lower than the dissolved concentrations in our MI study lakes.

The perch sperm did not seem to be highly sensitive to activation water-mediated, short-term exposure to water containing elevated concentrations of metals. From the tested activation waters, only the artificial freshwater spiked water with extremely high Cd concentration (50 mg L^−1^) reduced the proportion of motile sperm. Similar high tolerance of fish sperm to short-term exposure of several different metals has been observed earlier by Lahnsteiner et al. (2004) for different fish species.

In the contaminated study lakes, the measured characteristics of hepatic, somatic, and reproductive tissues of perch males have been altered under the multi-stressor conditions caused by increased metal concentrations and elevated salinity. The measured growth and condition variables are sensitive to many randomly or systematically varied within-lake and interlake factors, i.e., population dynamics of food organisms, internal annual cycles in the perch population dynamics, length of growing season in relation to annual phenology and water temperature, and other anthropogenic changes in the catchment area of the lakes. Although our research design includes only one reference lake of four lakes and each contaminated lakes with different contamination profile and other morph-edaphic features, the results suggested that condition and sperm measures of fish could be useful indicators for metal mining impacts on freshwater fish, but only if high natural interlake and phenological variation in these characteristics can be controlled by a multiyear monitoring design.

## Data Availability

The datasets generated during and/or analysed during the current study are available from the corresponding author on reasonable request.
